# Direct Micromachining of Microfluidic Channels on Biodegradable Materials Using Laser Ablation

**DOI:** 10.3390/polym9070242

**Published:** 2017-06-23

**Authors:** Yi-Kong Hsieh, Shiau-Chen Chen, Wen-Ling Huang, Kai-Ping Hsu, Kaiser Alejandro Villalobos Gorday, Tsinghai Wang, Jane Wang

**Affiliations:** 1 Department of Chemical Engineering, National Tsing Hua University, Hsinchu 30013, Taiwan; d944530@oz.nthu.edu.tw (Y.-K.H.); simple510285@gmail.com (S.-C.C.); e23376196@gmail.com (K.-P.H.); Kavg_9113@hotmail.com (K.A.V.G.); 2 Department of Biomedical Engineering and Environment Sciences, National Tsing Hua University, Hsinchu 30013, Taiwan; x123456789etgy@gmail.com (W.-L.H.); gominy01@gmail.com (T.W.)

**Keywords:** laser ablation, microfluidic fabrication, biodegradable polymeric material

## Abstract

Laser patterning on polymeric materials is considered a green and rapid manufacturing process with low material selection barrier and high adjustability. Unlike microelectromechanical systems (MEMS), it is a highly flexible processing method, especially useful for prototyping. This study focuses on the development of polymer surface modification method using a 193 nm excimer laser system for the design and fabrication of a microfluidic system similar to that of natural vasculatures. Besides from poly(dimethyl siloxane) (PDMS), laser ablation on biodegradable polymeric material, poly(glycerol sebacate) (PGS) and poly(1,3-diamino-2-hydroxypropane-*co*-polyol sebacate) (APS) are investigated. Parameters of laser ablation and fabrication techniques to create microchannels are discussed. The results show that nano/micro-sized fractures and cracks are generally observed across PDMS surface after laser ablation, but not on PGS and APS surfaces. The widths of channels are more precise on PGS and APS than those on PDMS. Laser beam size and channel depth are high correlation with a linear relationship. Repeated laser ablations on the same position of scaffolds reveal that the ablation efficiencies and edge quality on PGS and APS are higher than on PDMS, suggesting the high applicability of direct laser machining to PGS and APS. To ensure stable ablation efficiency, effects of defocus distance into polymer surfaces toward laser ablation stability are investigated. The depth of channel is related to the ratio of firing frequency and ablation progression speed. The hydrodynamic simulation of channels suggests that natural blood vessel is similar to the laser patterned U-shaped channels, and the resulting micro-patterns are highly applicable in the field of micro-fabrication and biomedical engineering.

## 1. Introduction

Micro- and nano-patterning of polymeric materials are commonly created by replica molding from silicon wafers produced from photolithography through MicroElectroMechanical Systems (MEMS) [1,2,3,4], allowing for high reproducibility of patterns with millimeters to submicron feature sizes. Replica molding of Poly(DiMethylSiloxane) (PDMS) from a silicon wafer master patterned with photoresist was first published by the Whitesides Laboratory in 1996 [5,6] and was popularly utilized for the production of micro- and nano-patterned PDMS films later on. The method was further adopted in the production of various patterned polymeric films from different polymers, i.e., Poly(Glycerol Sebacate)s (PGS) [7,8], 1,3-diamino-2-hydroxypropane-*co*-polyol sebacate (APS) [9], polyimides [10], Poly(Lactide-*co*-Glycolide) (PLGA) [11], polyethersulfone [12] … etc. Although MEMS remain one of the most popular fabrication methods in micro- and nano-scale, and are considered highly applicable toward the mass production of micro- and nano-scaled products, the high cost from mold fabrication together with difficulties in polymer delamination render it less than ideal for prototyping devices in similar scales.

Laser-based technology is considered green and convenient, and has been applied to fabricating devices with micro/submicro-scale features [13,14,15] and additive manufacturing (e.g., laser-induced forward transfer (LIFT) or laser sintering (LS) [16,17,18,19]. The unique energy source of laser is characterized by its spectral purity, spatial and temporal coherence, and high average peak intensity [20]. The mechanism between laser and material surface involves electronic excitation/de-excitation, melting, dissociation/decomposition (broken chemical bonds cause the material to dissociate/decompose), evaporation and material expulsion from the area of laser–material interaction [21,22,23]. The vital parameters governing this mechanism are laser wavelength, and material properties, e.g., reflectivity, thermal conductivity, specific heat and evaporation [22,24]. The materials with high absorption coefficients to specific laser wavelength and low thermal conductivities perform better ablation efficiency, and the thermal effects, such as melting, cracking, and burr formation, will be limited [25,26].

Laser direct writing/machining is a recent, flexible, fast and cost-effective approach in the production of micro/submicro-scale devices [27,28,29]. Unlike MEMS-Fab, mask fabrication for photolithography is no longer needed in laser direct writing techniques [30,31,32], and the designs or patterns can be modified in hours of notice. However, it has also been reported that the channels on Poly(methyl methacrylate) (PMMA) and PDMS created by laser direct machining usually come with bulges, splashes and distorted sidewalls [33,34,35,36]. Although there are some studies that overcame this shortage by various techniques, such as covering PDMS on glass materials [37], water-assisted laser ablation [38], and molding/remolding with PDMS [39], these steps are either laborious or time-consuming and have difficulties controlling channel depths. Despite the shortcomings, laser ablation showed great promises in polymeric scaffold fabrication and its applicability in various fields, especially tissue engineering [12,40,41]. However, very little attention had been paid to improving the fabrication quality and controllability. In this study, the details of the manufacturing of microfluidic channels using laser ablation as a fast and affordable alternative for MEMS were investigated. Through different laser beam size and repeated ablations, a full microfluidic system mimicking true blood vessel networks similar to the connection from arteries to capillaries and capillaries to veins can be fabricated in a fast and high definition manner.

Biodegradable polymers are commonly used in scaffold fabrication for tissue engineering. However, common biodegradable polymers, such as poly-L-lactic acid (PLLA), PLGA, polycaprolactone (PCL) ... etc., suffer from high Young’s modulus, and long degradation half-life, thus limiting the applicability to a portion of tissue engineering fields. PGS [42] and APS [43] are two glycerol-based elastomeric biodegradable polymers that show great promise in the field of soft tissue engineering for their low MPa range Young’s modulus and moderately fast degradation rate. Since PDMS is known to be biocompatible but not biodegradable and to have poor surface stability, PGS and APS are introduced in the hope of replacing PDMS. In 2008, the work of Engelmyer et al. first demonstrated the potential of laser-ablated elastomeric material in tissue engineering, revealing the micromachining need in soft tissue engineering [44]. In this work, to further investigate the applicability of PGS and APS in vasculature regeneration, laser ablation is applied on the fabrication of microfluidic systems from PDMS and two biodegradable materials, PGS and APS. The methodologies of direct writing on polymer films using an excimer laser were developed, and the relationship between laser parameters and ablation efficiency were thoroughly studied. In particular, the effect and the potential limitations of laser fluence, beam size, repeated ablation, firing frequency, ablation progression speed, and defocus distance were examined and characterized. The applicability of laser ablation to PGS and APS polymers processing are proven an effective and precise micro-fabrication method.

## 2. Materials and Methods

### 2.1. PGS Synthesis

All materials were purchased from Aldrich and used as received unless otherwise specified. The PGS pre-polymer was synthesized by step growth polymerization of 0.1 mole each of glycerol (Aldrich, St. Louis, MO, USA) and sebacic acid heated under a nitrogen blanket at 130 °C for 24 h according to previously published methods [42]. The resulting product was stored under a desiccant environment as is for later use. The product was spread onto silicon wafer molds and glass slides and cured at 160 °C at approximately 10^−3^ Torr for 24 h.

### 2.2. APS Synthesis

The synthesis of APS was carried out as described by Bettinger et al. [43] with slight modification of the reaction parameters. A round-bottom flask was charged with 0.06 mol of 1,3-diamino-2-hydroxy-propane (DAHP), 0.03 mol glycerol (G) and 0.09 mol of sebacic acid (SA) to produce a molar ratio of 2:1:3 of DAHP:G:SA, respectively. The reactants were heated under a nitrogen blanket at 130 °C. The pressure was then dropped to approximately 10^−3^ Torr and the contents were allowed to react for 24 h. The product was then stored under a desiccant environment until further use. The product was spread onto silicon wafer molds and glass slides and cured at 180 °C at approximately 10^−3^ Torr for 24 h. Film thicknesses of 1 mm were achieved.

### 2.3. PDMS Synthesisar

The PDMS oligomer and crosslinking prepolymer of PDMS agent from a SylgardTM 184 kit (Dow and Corning, Auburn, MI, USA) was mixed in a weight ratio of 10:1 and cast in Petri dishes. After a degas process, PDMS layers with height of approximately 0.1 mm were cured at 65 °C for 24 h.

### 2.4. Laser Ablation System

The channels of pattern were ablated by a New Wave UP193 ArF excimer laser system (Electro Scientific Industries, San Jose, CA, USA). In this study, the working parameters for laser ablation are listed in Table 1. Briefly, the laser wavelength was 190 nm and pulse width 4 ns; ablation progression speed of 100 μm s^−1^ was used; frequency of laser shots was 10 Hz; the laser energy was between 6–9 J cm^−2^; beam sizes of 10–150 μm were chosen, and the repeated ablations were conducted for 1, 10, 20, 40, 60, 80 and 100 times in this study. During the ablation, the defocus distances were set as 0 (focus), 300, 500, 800 and 1000 μm. The sample was positioned in the ablation cell and could be translated by a computer-controlled XYZ stage to the focused laser beam.

### 2.5. Surface Topography Measurement

Surface topography profilometer (Bruker corp., Billerica, MA, USA) is an advanced thin measurement tool detecting the sample beneath a diamond-tipped stylus. The high-precision stage moves a sample beneath the stylus according to a user-programmed scan length, speed and stylus force. In this work, 2000 μm of scan length, 50 μm s^−1^ of speed, stylus tip size of 2 μm and 10 mg of stylus force were used. The depth of channels in this work was measured by surface topography measurement unless otherwise specified.

### 2.6. Field Emission Scanning Electron Microscope (FE-SEM) Image

Field emission scanning electron microscope (Hitach, Tokyo, Japan) was used to measure the channel depth which is created by repeated ablation. The samples were prepared by sputter coating with palladium for 90 s. Images were taken under 5 and 10-kV accelerating voltages.

### 2.7. Fabrication of Microfluidic Device

A polyethylene tube with inner diameter of 580 μm (Becton Dickinson, San Jose, CA, USA) was glued to a petri dish, and both ends of it were sealed. PDMS prepolymer mixed with crosslinking agent was cast in the petri dish until the entire tube was immersed. After degassing and curing at 65 °C for 24 h, the tube-embedded PDMS was used as the base layer of the flow device. Oxygen plasma etcher (Advanced Research Technology, Hsinchu, Taiwan) was set with oxygen flow rate at 20 cm^3^ min^−1^ for 1 min, chamber pressure at 500 mTorr, and power at 100 W to attach PGS/APS to the PDMS base layer. The sample was ablated by laser system, and the debris in the patterns on PDMS was removed. A connecting channel was created between the PGS/APS patterns and the tubes embedded in PDMS via laser ablation, and the pattern was covered with a thin PDMS/PGS/APS film as described in previous study [9]. Methylene blue solution was used to visualize the flow in microfluidic channels.

### 2.8. Hydrodynamics Simulation of Micropatterns

In this work, the COMSOL Multiphysics^®^ Modeling Software (5.1, COMSOL Inc., Burlington, MA, USA, 2016) was applied in hydrodynamic simulation of velocity in the micro-channels. The viscosity of fluid and hydraulic diameter were calculated:

(1) $$\frac{Q}{A}=v,$$

(2) $$D_{h}=\frac{4\times A}{p},$$

In Equation (1), flow velocity (*v*) is equal to the ratio of flow rate (*Q*) and cross-sectional area (*A*). The cross-sectional area of squared, U-shaped and circular channels is 2.25 × 10^−8^, 1.91 × 10^−8^ and 1.77 × 10^−8^ μm^2^, respectively. The hydraulic diameter, *D_h_*, can also be calculated by the ratio of four times of cross-sectional area (*A*) and wetted perimeter of the cross-section (*p*) (Equation (2)). The wetted perimeter of the cross-section of squared, U-shaped and circular channels is 600, 527 and 471 μm, respectively. The hydraulic diameter can be calculated as 150, 150 and 145 μm for squared, U-shaped and circular channels, and the flow velocity is 1.2 × 10^−2^, 8.7 × 10^−3^, and 9.5 × 10^−3^ ms^−1^.

When using water as the working medium, the density (ρ) was 998.2 kg m^−3^ and dynamic viscosity (*η*) was 9.38 × 10^−4^ Ns m^−2^ at 25 °C working temperature. The Reynolds number (Re) was calculated as shown in Equation (3):

(3) Re=pvDhη,

The Reynolds number of squared, U-shaped and circular channels was found to be 1.9, 1.3 and 1.5, respectively. The entrance length ($l_{\theta }$) is calculated by Equation (4):

(4) lθ=0.0125×Re×Dh,

To reach the fully developed laminar flow, the entrance length for squared, U-shaped and circular channels is 3.5 × 10^−6^, 2.4 × 10^−6^, and 2.8 × 10^−6^ m, respectively. In this study, the flow rates are simulated at 0.05, 0.1 and 0.2 μL min^−1^.

## 3. Results and Discussions

### 3.1. Influence of Laser Fluence toward Polymer Patterning

**Figure 1:**
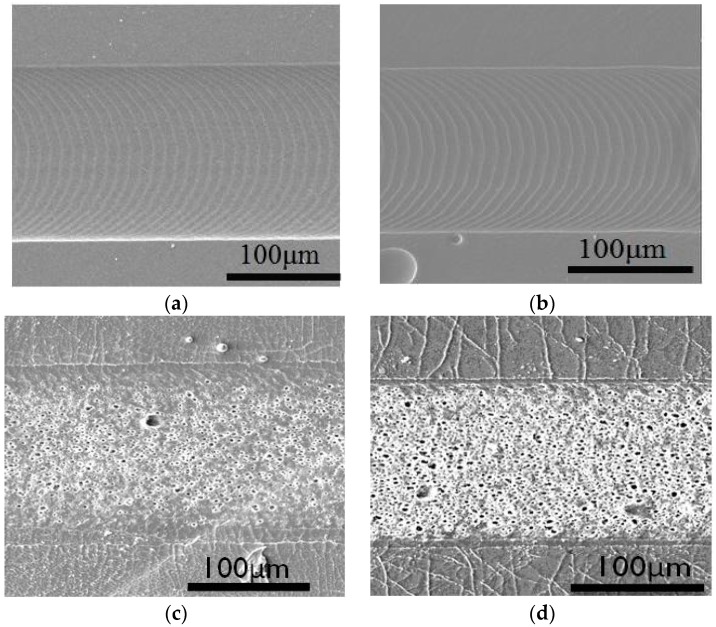
Scanning Electron Microscope (SEM) images of (**a**) poly(glycerol sebacate) (PGS) ablated under laser fluence of 6 J cm^−2^; (**b**) poly(1,3-diamino-2-hydroxypropane-*co*-polyol sebacate) (APS) ablated under laser fluence of 6 J cm^−2^; (**c**) poly(dimethyl siloxane) (PDMS) ablated under laser fluence of 6 J cm^−2^; (**d**) PDMS ablated under laser fluence of 9 J cm^−2^.

In order to create micro-patterns on polymeric surface via laser ablation, the influences of laser fluence to ablation depth on polymeric materials were examined. In Figure 1, SEM images of PGS, APS and PDMS ablated at 6 J cm^−2^ are shown, along with PDMS ablated at 9 J cm^−2^. At 6 J cm^−2^, there are clear channels with microgrooves from laser ablation on both PGS and APS films. In contrast, channels with cracked edges together with rough and porous surfaces along with charred pieces are observed on PDMS at 9 J cm^−2^. PDMS ablated at higher energy (9 J cm^−2^) are shown in Figure 1d. More fractures are found in Figure 1d than in Figure 1c, and SEM images clearly indicate that these nano/micro-sized fractures are present along the channels and the density of cavities inside the ablated channels increased with increasing ablation energy. Observation of ablated channels indicates that though the depth of the channels remains steady across different laser fluence, the edge quality of the channels varies widely. In order to create comparable microchannels on PDMS, PGS and APS, it is concluded that the laser fluence capacity used in this study will be set at 6 J cm^−2^ to avoid creating cracked and burnt PDMS channels.

It is worth noting that these fractures are not only present in the surface of channels, but inside the polymeric materials in a radially out fashion. It is believed that the different ablation efficiency across different materials and the presence of nano/micro-sized fractures were results of a combined effect of two factors: the thermal conductivity of polymeric materials and the total energy received from higher laser fluence. As PDMS was less thermally conductive, high energy was accumulated at the surface of the polymer, creating shallow channels with cracks and burns around the channel. As APS and PGS were both materials synthesized via step growth polymerization under high temperature and low pressure, laser energy conducted and dissipated evenly through a thick surface while maintaining smooth channel surfaces.

### 3.2. Control of Channel Widths and Depths

As the width of channels created via laser ablation depended solely on the size of aperture in a laser ablation system, it is considered the key factor in creating bifurcated networks mimicking blood vessels. Table 2 lists the measured widths of channels created under various beam sizes ranging from 10 μm to 150 μm on PGS, APS and PDMS. It is clear that the accuracy of laser ablation decreased slightly with increasing beam size, but was overall very stable. Overall, it is clear that the channel width created via laser ablation is easily manipulated with high accuracy.

**Figure 2:**
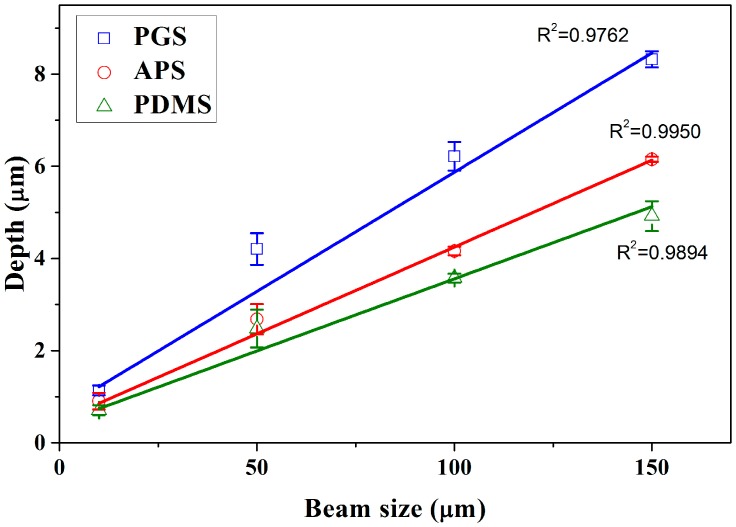
The variations of measured ablation depth using beam sizes of 10, 50, 100 and 150 μm at ablation progression speeds of 100 μm s^−1^, frequency of 10 Hz and energy of 6 J cm^−2^ to ablate a single channel on PGS, APS and PDMS (*n* = 3).

Through the modification in beam sizes (aperture size), the width of microchannels is highly tunable, and possesses a wide application, especially in mimicking microvasculature. For example, by utilizing beam size of 10 μm, it is possible to create channels resembling capillary, while 50 μm beams are capable of mimicking the arteriole and venule. Through theoretical calculation, at constant fluence, as the beam sizes increases, the total energy per ablation increases linearly with area. This effect was observed in Figure 2, which measures the variations in depth over different beam sizes (obtained from Surface Topography Profilometer) on PGS, APS and PDMS under fixed ablation fluence 6 J cm^−2^. The depth is observed to have increased with beam size, and the coefficients of beam size with depth on PGS, APS and PDMS were 0.9762, 0.9950 and 0.9894, respectively. As discussed in the work of Sizyuk et al. [45], increase in beam sizes (spot sizes) often lead to higher plasma temperature, which may directly result in higher etching efficiency.

**Figure 3:**
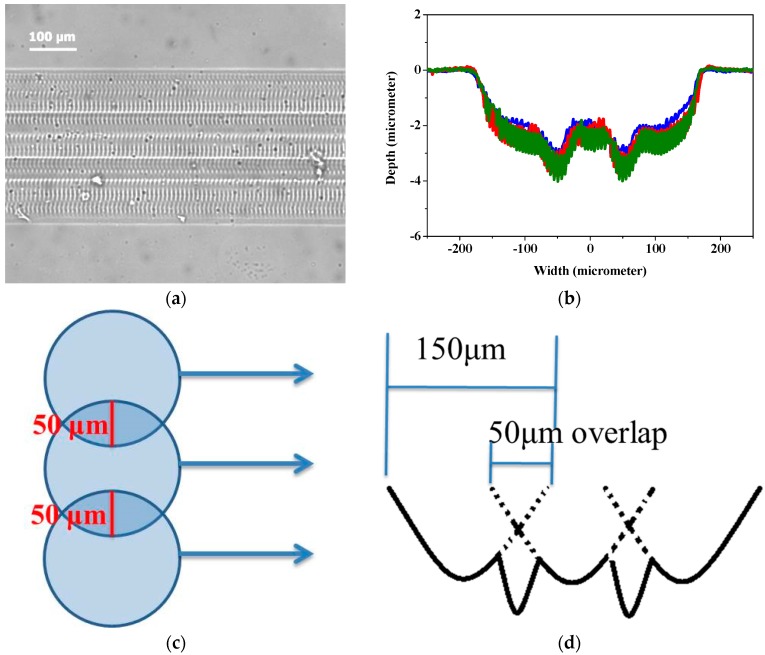
(**a**) Microscope photograph, (**b**) laser ablation shape, (**c**) schematic diagram of overlapping effect and (**d**) schematic diagram of channel ablated by overlapped pattern. The conditions of lasers: fluence of 6 J cm^−2^, beam size of 150 μm, beam firing frequency at 10 Hz, ablation progression speed at 100 μm s^−1^, and ablated once.

The width of ablated channels is directly correlated to the beam size of laser used, and in this work, it ranged between 1 to 150 μm. In order to create wider channels, channels created by overlapping linear laser ablation were investigated. In Figure 3, 350 μm wide channels were created with three linear ablations operated with beam size of 150 μm, fluences of 6 J cm^−2^, ablation frequency at 10 Hz and ablation progression speed at 100 μm s^−1^. Each linear pattern overlapped by 50 μm, and the total width of three ablations with two overlaps was about 350 μm. In Figure 3a, a linear channel of 350 μm wide with two visible overlapped sections are observed under optical microscope. Surface profilometer was employed to further examine the depth of channel made by overlapped patterns, and is shown in Figure 3b. The results show that there are two dimples in the bottom of channels, creating a “w” shaped pattern. It is verified that the position of the dimples in the channel is consistent with the overlapped area of each linear pattern (Figure 3c). As a result, it may suggest that the two dimples were caused by overlapped ablation, as shown in Figure 3d. The overlapped area was doubly ablated, thus received more energy than other parts, resulting in the two dimples. It is noted that there are micropatterns inside the channels shown in Figures 1a,b and 3a perpendicular to the moving direction of laser beam, but they are relatively shallow compared to the channels, such that they were not observed through the surface profilometer at all, as shown in Figure 3b.

### 3.3. Repeated Ablation to Vary Channel Depth

**Figure 4:**
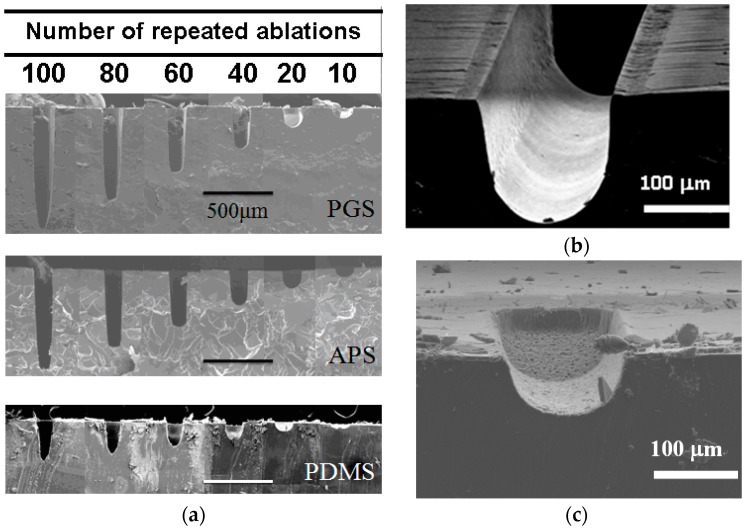
(**a**) The SEM images of three materials with increasing ablation times. (**b**) Laser ablated 25 times on PGS presented high edge quality. (**c**) Laser ablated 25 times on PDMS presented poor edge quality.

The channel depths of the microchannels are highly tunable by repeated ablation on scaffold. As shown through SEM imaging in Figure 4a, repeated laser ablation on the same position of scaffold created indentations of deeper depth. Good edge quality is observed in PGS in Figure 4b. This is likely due to the thermal stability previously discussed, where the energy from laser source was dissipated evenly into surrounding material instead of accumulating around the pattern creating debris as observed in PDMS (Figure 4c).

**Figure 5:**
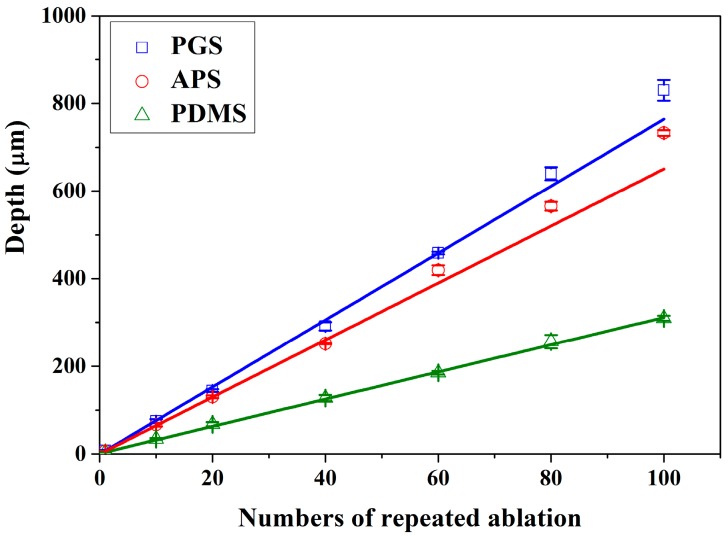
Number of repeat ablations and depth of channels are in direct correlation with slope of 7.64, 6.51 and 3.11 μm per ablation for PGS, APS and PDMS, respectively.

The changes in depth are linearly proportional to the number of repeated ablation as shown in Figure 5, with ablation efficiency of 7.64, 6.51 and 3.11 μm per ablation on PGS, APS and PDMS, respectively. As observed above, the ablation efficiency is the highest in PGS and lowest in PDMS, indicating that there may be more precise control over the depth of channels ablated on PDMS, though the edge quality might be slightly lower. Meanwhile, it is most efficient to create patterns using laser ablation on PGS. Overall, laser ablation is capable of creating channels of any depth by controlling the numbers of repeated ablation, easily changing the aspect ratio of any pattern.

Through the analyses in Figure 1, Figure 2, Figure 3, Figure 4 and Figure 5, it is found that even though PDMS is one of the most commonly used materials in the fabrication of microfluidic devices, it is also the least thermally stable amongst the three, thus less susceptible toward laser ablation. APS and PGS both are thermally stable under laser ablation, and PGS is especially useful as a laser ablation material due to its high laser ablation efficiency and high edge quality. Therefore, for the remaining studies, PGS was chosen as the main material of investigation.

### 3.4. The Relation between Defocus Distance and Ablation Efficiency

**Figure 6:**
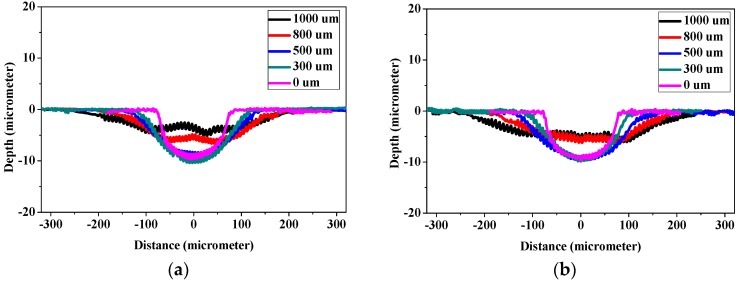

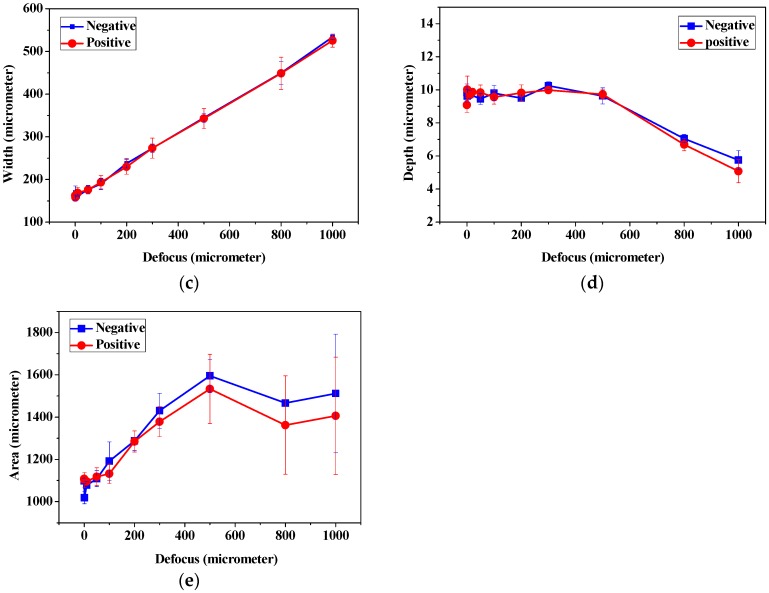
Laser ablation shape at different defocus (**a**) negative and (**b**) positive. The corresponding (**c**) width, (**d**) depth and (**e**) area of channels ablated at different distances of defocus. The conditions of lasers: fluence of 6 J cm^−2^, beam size of 150 μm, beam firing frequency at 20 Hz, ablation progression speed at 100 μm s^−1^, and 1 time of repeated ablation.

With the direct writing capacity of laser ablation, laser beams were adjusted along the *x*- and *y*-axes to create microchannels, while repeated ablation enabled the deepening of channels along the *z*-axis. However, further investigation by adjusting laser focal point along the *z*-axis was considered vital, since laser focal point offsets may result in defocusing of laser beam during the laser patterning process. In Figure 6, the laser with beam size of 150 μm, fluence of 6 J cm^−2^, ablation frequency at 20 Hz and ablation progression speed of 100 μm s^−1^ was employed to create channels on PGS scaffolds at focus (0 μm) and defocused at 300, 500, 800 and 1000 μm each. The cross-sectional depth and width were measured with surface profilometer and the area was integrated over the measured depth and width. Figure 6a clearly illustrates the increase in width and decrease in depth as laser beams become more negatively defocused, while Figure 6b illustrates the result of positively defocused laser beams. It is clear that the two figures are nearly identical, indicating that when laser beam is defocused from the surface of the polymer surface, positive and negative defocus are equivalent in polymer removal rate. Further investigation of the effect of laser defocus on depth of ablation is shown in Figure 6c, the width of channel increased with the defocus distances, and this phenomenon depends on the size of plasma formed by the laser shot, which is determined by defocus distance of laser from the surface of substrate. A proportional linearity is thus expected. Figure 6d depicts the ablation depth created at different defocus distances. It is observed to remain steady around 10 μm per ablation within 500 μm of defocus, and linearly decreased beyond 500 μm. On the other hand, the width of the channels increased with increasing defocus distance proportionally. Thus the depth of channels were affected by the energy dissipation of plasmas to the substrates at constant fluence, and energy density of the plasmas dropped significantly for larger defocus distance, resulting in reductions in ablated depth and area (Figure 6e).

By combining the observations in Figure 6d,e, it is clear that for reliable ablation depth estimation, the defocus distance must be readjusted within depth of 500 μm. However, since Figure 6c clearly illustrates that the width of channels increases with defocus distance linearly, the error margin for width may be well over 200%, making it essential to readjust the defocus distance earlier than 500 μm. In this work, it is suggested that the defocus distance be adjusted every 75 μm ablated in depth, so that the variation in channel width be maintained within 20%.

### 3.5. The Influence of Laser Firing Frequency and Ablation Progression Speed to Channel Depth

**Figure 7:**
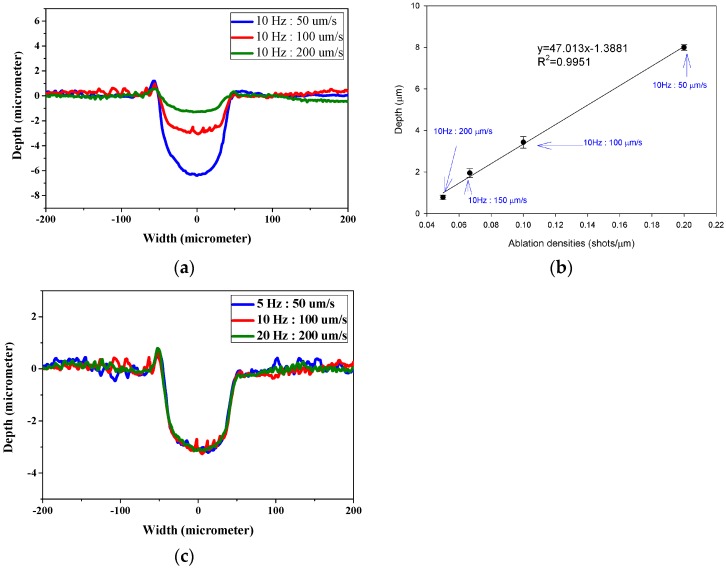
(**a**,**c**) Laser ablation shape and (**b**) the corresponding depth channels ablated at different ratios between ablation frequency and ablation progression speed. Laser was operated with fluence of 6 J cm^−2^ and 150 μm beam size. The ratio between firing frequency and ablation progression speed is as indicated.

Figure 7 demonstrates the laser ablation shape and corresponding depth of channels ablated at different ratios between firing frequency and ablation progression speed. In Figure 7a, the channels ablated at ablation progression speed of 100 μm s^−1^ appear almost doubled in depth than that of 200 μm s^−1^ when the firing frequency is 10 Hz, indicating an inverse correlation between channel depth and ablation progression speed. The corresponding depth of channels ablated at different ratios between firing frequency and ablation progression speed are presented in Figure 7b. The concept of ablation densities along the moving axis is proposed by calculating the total number of ablations occurring in 1 μm distance. The results suggest that the higher the ablation density, the deeper the depth of channels. This indicates a direct result of increased accumulation of energy per ablation. With the high correlation (R^2^ = 0.9951), it is concluded that channel depth is inversely correlated to ablation progression speed. As the ablation progression speed and beam firing frequency is adjusted, it is hypothesized that the similar “ratio” between the two is a more dominant factor than the absolute numbers. To analyze from an ablation per area perspective, a laser beam firing at 10 Hz and moving at 100 μm s^−1^ fires the same number of shots as one ablating at 20 Hz, 200 μm s^−1^. The main difference between the two is that the amount of energy received in the same amount of time is doubled. As shown in Figure 7c, the ablation shape made by the different ablation progression speed and beam firing frequency is the same regardless of the absolute ablation progression speed and beam firing frequency. This phenomenon is also observed by Arutinov et al. with respect to nanosecond and picosecond lasers [16].

### 3.6. Hydrodynamic Simulation of Laser Patterned Channels

As described in Figure 4a–c, microfluidic channels created with laser ablation are generally U-shaped. In order to understand the difference between the laser-ablated micro-channels and natural blood vessels, an analysis using COMSOL hydrodynamic simulation is performed. Three shapes of channels were chosen for the hydrodynamic simulation: squared, U-shaped and circular cross-sectional channels as shown in Table 3. These three shapes were chosen as square and U-shaped channels share the linear top edge, while circular channels are most similar to that of the natural blood vessels. The simulation results suggest that geometric variations of microchannels present nearly no influence on the hydrodynamics except for the maximum velocity. The maximum velocity of the flux was 156, 180 and 191 μm s^−1^ in squared, U-shaped and circular channels, respectively, when flow rate is fixed at 0.1 μL min^−1^. The maximum velocity between U-shaped and circular channels was different by 6.11%, but the maximum velocities between U-shaped and squared channels were different by 13.33%. This suggests that the performance of channels made by laser ablation (U-shaped) is more similar to that of natural blood vessel (circle) than a squared channel, and the assembly process of the U-shaped microfluidic device is very similar to those fabricated via MEMS-Fab.

### 3.7. The Fabrication of Simple Microfluidic Systems via Laser Ablation

**Figure 8:**
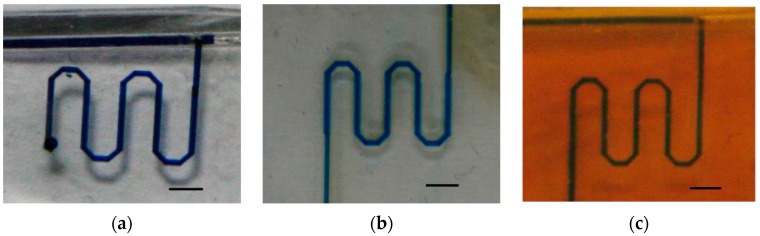

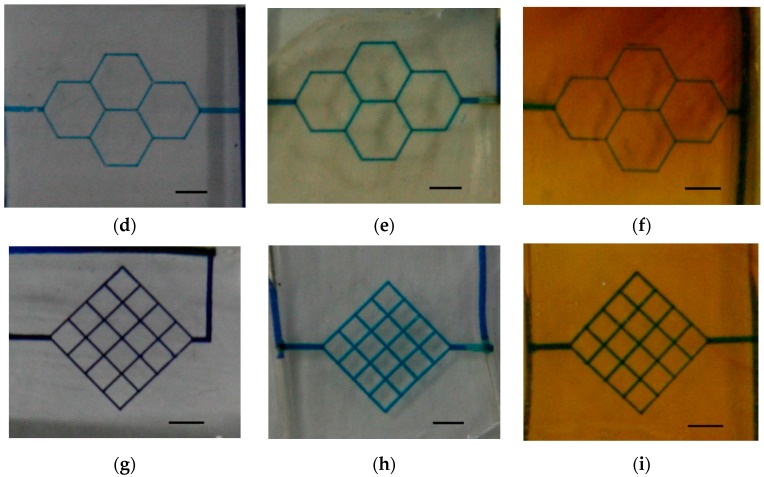
Microfluidic devices with (**a**) single-channel pattern on PDMS; (**b**) honeycomb pattern on PDMS; (**c**) mesh-like pattern on PDMS; (**d**) single-channel pattern on PGS; (**e**) honeycomb pattern on PGS; (**f**) mesh-like pattern on PGS; (**g**) single-channel pattern on APS; (**h**) honeycomb pattern on APS; (**i**) mesh-like pattern on APS. The scale bar is 0.5 cm.

Three microfluidic patterns—single-channels, honeycombs and mesh-like patterns—were prototyped using laser ablation on PDMS, PGS and APS, as shown in Figure 8. The microfluidic device with single-channel patterns may serve as micro-reactor, and consume less reagent and sample than bulk systems [46]. The honeycombs and mesh-like patterns were adapted from studies of biomaterial scaffolds [47,48], which proved that laser ablation is useful in creating micropatterns. In this study, patterns were tested at 0.1 μL min^−1^ for a minimum of 24 h, and a minimum of three flow experiments were repeated to prove the robustness and repeatability. The tests of flow in the channels are displayed in the animation (Video S1). However, it should be mentioned that the flows in the microfluidic patterns on PDMS were blocked more often than those on PGS and APS due to the excess debris in PDMS channels, largely due to the poor edge quality from laser ablation.

## 4. Conclusions

In this study, laser ablation was applied as a simple and convenient method for the fabrication of microfluidic flow devices. By removing the steps of masks/molds production in photolithographic or soft photolithographic technique, this fabrication method holds great potential as a prototyping method. The relations of ablated width as well as depth-to-laser parameters were investigated, and the relations between laser energy and surface changing on the polymeric materials were examined. The desired depth of pattern was adjusted by repeated ablations, and accompanied by defocus adjustments, as it was found that the defocus distance affects the channel width and depth, significant error when larger than 500 μm. Therefore, adjustment in focus is suggested to be made every 75 μm for stable ablation efficiency. Further investigation toward depth manipulation using laser ablation also indicated that ablation density based on the combination of firing frequency and ablation progression speed is another factor in controlling laser ablation efficiency. Although traditional MEMS-Fab is well known for high-precision manufacturing of microchannels with a wide range of width, it is highly limited by the photolithographic property toward depth variation within the same mold. Meanwhile, one common obstacle in reverse molding of polymeric material is the challenge in delaminating the patterned films without damaging the micro/nanopatterns. Instead, flat polymeric films, which are much easier to produce, are employed after delamination in direct writing using laser ablation, greatly reducing the risk of damaging the patterns. Through the application of laser ablation, wide and deep channels can be produced alongside narrow and shallow channels in one simple process, creating microfluidic systems that are much more analogous to natural vasculatures.

Three prototypes of microfluidic designed in this study were revealed, and channel patterns were created on the polymeric materials for modular bio-analytical chip applications. Using laser ablation, device fabrication and prototyping were fast and precise. The technique may become useful in analyzing liquid sample, simulating blood vessels and sample pretreatment or mixing device. It is proven that laser ablation is an effective method for the creation of microfluidic devices with micro- even nano-sized to mimic the blood vessels. The reproducibility and quality of devices are excellent for biological and chemical researches. Simulation suggested that the performance of channels made by laser ablation is similar to that of natural blood vessel. By carefully controlling the parameters of laser, high-precision micro-channels without cracks/debris were produced. The predictable ablation performances on PGS as well as APS reveal the potential in application of ArF laser in the microfabrication of polymeric materials and bioengineering studies. However, much similar to the result of predecessors, PDMS is again proven a poor material of choice for laser ablation due to its low thermal conductively and poor edge quality.

## Figures and Tables

**Table 1:** Operation conditions for laser ablation.

Parameter	Value
Laser wavelength	193 nm
Pulse Width	4 ns
Ablation progression speed	50–200 μm s^−1^
Frequency of laser shots	5–20 Hz
Laser energy	6 and 9 J cm^−2^
Beam size	10–150 μm
Repeated ablations	1–100 times
Defocus distance	0–1000 μm

**Table 2:** The widths of linear channels ablated on PGS, APS, and PDMS ablated with 10 μm, 50 μm, 100 μm, and 150 μm beam size at ablation progression speed of 100 μm s^−1^, frequency of 10 Hz and energy of 6 J cm^−2^. The measured widths are obtained through α-step. All units are in micrometers.

Beam Size	PGS	APS	PDMS
10	8 ± 1	11 ± 2	12 ± 2
50	48 ± 3	52 ± 3	52 ± 3
100	97 ± 5	104 ± 6	102 ± 6
150	156 ± 9	157 ± 7	157 ± 7

**Table 3:** Hydrodynamics simulations of different shapes microchannel (Scale unit is ms^−1^).

Flow Rate (Q)	0.05 μL min^−1^	0.1 μL min^−1^	0.2 μL min^−1^	
Squared	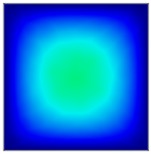	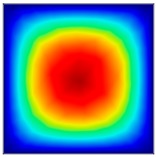	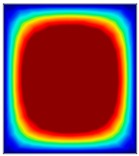	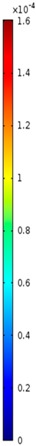
U-shaped	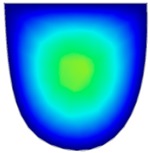	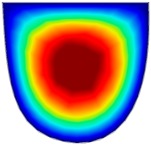	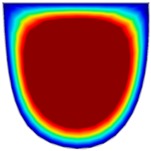	
Circular	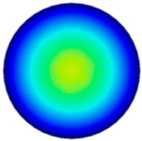	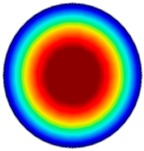	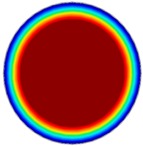	

## Supplementary Materials

The following are available online at www.mdpi.com/2073-4360/9/7/242/s1, Video S1: the flow in the proposed microfluidic device.

## References

[1] Bong-Hwan K., Jong-Bok K. (2009). Fabrication of a high aspect ratio thick silicon wafer mold and electroplating using flipchip bonding for mems applications. J. Micromech. Microeng..

[2] Marasso S.L., Canavese G., Cocuzza M. (2011). Cost efficient master fabrication process on copper substrates. Microelectron. Eng..

[3] Ho C.-H., Chin K.-P., Yang C.-R., Wu H.-M., Chen S.-L. (2002). Ultrathick su-8 mold formation and removal, and its application to the fabrication of liga-like micromotors with embedded roots. Sens. Actuators A Phys..

[4] Chang H.-K., Kim Y.-K. (2000). Uv-liga process for high aspect ratio structure using stress barrier and c-shaped etch hole. Sens. Actuators A Phys..

[5] Xia Y., Whitesides G.M. (1998). Soft lithography. Angew. Chem. Int. Ed..

[6] Xia Y., Kim E., Zhao X.-M., Rogers J., Prentiss M., Whitesides G. (1996). Complex optical surfaces formed by replica molding against elastomeric masters. Science.

[7] Fidkowski C., Kaazempur-Mofrad M.R., Borenstein J., Vacanti J.P., Langer R., Wang Y. (2005). Endothelialized microvasculature based on a biodegradable elastomer. Tissue Eng..

[8] Bettinger C.J., Weinberg E.J., Kulig K.M., Vacanti J.P., Wang Y., Borenstein J.T., Langer R. (2005). Three-dimensional microfluidic tissue-engineering scaffolds using a flexible biodegradable polymer. Adv. Mater..

[9] Wang J., Bettinger C.J., Langer R.S., Borenstein J.T. (2010). Biodegradable microfluidic scaffolds for tissue engineering from amino alcohol-based poly(ester amide) elastomers. Organogenesis.

[10] Cao Y., Zhou L., Wang X., Li X., Zeng X. (2009). Micropen direct-write deposition of polyimide. Microelectron. Eng..

[11] King K.R., Wang C.C.J., Kaazempur-Mofrad M.R., Vacanti J.P., Borenstein J.T. (2004). Biodegradable microfluidics. Adv. Mater..

[12] Brayfield C.A., Marra K.G., Leonard J.P., Tracy Cui X., Gerlach J.C. (2008). Excimer laser channel creation in polyethersulfone hollow fibers for compartmentalized in vitro neuronal cell culture scaffolds. Acta Biomater..

[13] Holmes A.S., Saidam S.M. (1998). Sacrificial layer process with laser-driven release for batch assembly operations. J. Microelectromech. Syst..

[14] Ke K., Hasselbrink E.F., Hunt A.J. (2005). Rapidly prototyped three-dimensional nanofluidic channel networks in glass substrates. Anal. Chem..

[15] Costa L., Terekhov A., Rajput D., Hofmeister W., Jowhar D., Wright G., Janetopoulos C. (2011). Femtosecond laser machined microfluidic devices for imaging of cells during chemotaxis. J. Laser Appl..

[16] Arutinov G., Mastrangeli M., Smits E.C.P., van Heck G., den Toonder J.M.J., Dietzel A. (2015). Foil-to-foil system integration through capillary self-alignment directed by laser patterning. J. Microelectromech. Syst..

[17] Gari A., Edsger C.P.S., Massimo M., Arutinov G., Smits E.C.P., Mastrangeli M., van Heck G., van den Brand J., Schoo H.F.M., Dietzel A. (2012). Capillary self-alignment of mesoscopic foil components for sensor-systems-in-foil. J. Micromech. Microeng..

[18] Römer G.R.B.E., Cerro D.A.D., Pohl R., Chang B., Liimatainen V., Zhou Q., Veld A.J.H.I. (2012). Picosecond laser machining of metallic and polymer substrates for fluidic driven self-alignment. Phys. Procedia.

[19] Schmid M., Wegener K. (2016). Additive manufacturing: Polymers applicable for laser sintering (ls). Procedia Eng..

[20] Lippert T., Lippert T.K. (2004). Laser application of polymers. Polymers and Light.

[21] Dutta Majumdar J., Manna I. (2003). Laser processing of materials. Sadhana.

[22] Jansen E.D., Frenz M., Kadipasaoglu K.A., Pfefer T.J., Altermatt H.J., Motamedi M., Welch A.J. (1997). Laser–tissue interaction during transmyocardial laser revascularization. Ann. Thorac. Surg..

[23] Mangirdas M., Holger G., Albertas Ž., Vytautas P., Domas P., Roaldas G. (2010). A femtosecond laser-induced two-photon photopolymerization technique for structuring microlenses. J. Opt..

[24] Samant A.N., Dahotre N.B. (2009). Laser machining of structural ceramics—A review. J. Eur. Ceram. Soc..

[25] Miller P., Aggarwal R., Doraiswamy A., Lin Y., Lee Y.-S., Narayan R. (2009). Laser micromachining for biomedical applications. JOM.

[26] Korte F., Nolte S., Chichkov B.N., Bauer T., Kamlage G., Wagner T., Fallnich C., Welling H. (1999). Far-field and near-field material processing with. Femtosecond laser pulses. Appl. Phys. A.

[27] Liao Y., Song J., Li E., Luo Y., Shen Y., Chen D., Cheng Y., Xu Z., Sugioka K., Midorikawa K. (2012). Rapid prototyping of three-dimensional microfluidic mixers in glass by femtosecond laser direct writing. Lab Chip.

[28] Jensen M.F., Noerholm M., Christensen L.H., Geschke O. (2003). Microstructure fabrication with a CO_2_ laser system: Characterization and fabrication of cavities produced by raster scanning of the laser beam. Lab Chip.

[29] Wagner F., Hoffmann P. (2000). Novel structure formation in poly(ethylene therephthalate) by scanning excimer laser ablation. Appl. Surf. Sci..

[30] Khan Malek C. (2006). Laser processing for bio-microfluidics applications (part i). Anal. Bioanal. Chem..

[31] Sugioka K., Hanada Y., Midorikawa K. (2007). 3d integration of microcomponents in a single glass chip by femtosecond laser direct writing for biochemical analysis. Appl. Surf. Sci..

[32] Sugioka K., Cheng Y. (2014). Ultrafast laser-reliable tools for advance materials processing. Light Sci. Appl..

[33] Fu L.-M., Ju W.-J., Yang R.-J., Wang Y.-N. (2013). Rapid prototyping of glass-based microfluidic chips utilizing two-pass defocused CO_2_ laser beam method. Microfluid. Nanofluid..

[34] Klank H., Kutter J.P., Geschke O. (2002). CO_2_-laser micromachining and back-end processing for rapid production of pmma-based microfluidic systems. Lab Chip.

[35] Li M., Li S., Wu J., Wen W., Li W., Alici G. (2012). A simple and cost-effective method for fabrication of integrated electronic-microfluidic devices using a laser-patterned pdms layer. Microfluid. Nanofluid..

[36] Liu H.-B., Gong H.-Q. (2009). Templateless prototyping of polydimethylsiloxane microfluidic structures using a pulsed CO_2_ laser. J. Micromech. Microeng..

[37] Chung C.K., Lin S.L., Chang K.C., Wang H.Y. Fabrication and simulation of pdms assisted CO_2_ laser ablation. Proceedings of the 2010 5th IEEE International Conference on Nano/Micro Engineered and Molecular Systems (NEMS).

[38] Chen C.-C., Lin P.-H., Chung C.-K. (2014). Microfluidic chip for plasma separation from undiluted human whole blood samples using low voltage contactless dielectrophoresis and capillary force. Lab Chip.

[39] Ziya I., Guler M.T., Berkan A., Ismail B., Caglar E. (2016). Rapid fabrication of microfluidic pdms devices from reusable pdms molds using laser ablation. J. Micromech. Microeng..

[40] Wang H.-W., Cheng C.-W., Li C.-W., Chang H.-W., Wu P.-H., Wang G.-J. (2012). Fabrication of pillared plga microvessel scaffold using femtosecond laser ablation. Int. J. Nanomed..

[41] Zainuddin, Chirila T.V., Barnard Z., Watson G.S., Toh C., Blakey I., Whittaker A.K., Hill D.J.T. (2011). F2 excimer laser (157 nm) radiation modification and surface ablation of phema hydrogels and the effects on bioactivity: Surface attachment and proliferation of human corneal epithelial cells. Radiat. Phys. Chem..

[42] Wang Y., Ameer G.A., Sheppard B.J., Langer R. (2002). A tough biodegradable elastomer. Nat. Biotechnol..

[43] Bettinger C.J., Bruggeman J.P., Borenstein J.T., Langer R.S. (2008). Amino alcohol-based degradable poly(ester amide) elastomers. Biomaterials.

[44] Engelmayr G.C., Cheng M., Bettinger C.J., Borenstein J.T., Langer R., Freed L.E. (2008). Accordion-like honeycombs for tissue engineering of cardiac anisotropy. Nat. Mater..

[45] Tatyana S., Ahmed H. (2014). Scaling mechanisms of vapour/plasma shielding from laser-produced plasmas to magnetic fusion regimes. Nucl. Fusion.

[46] Voiculescu I., Li F., Liu F., Zhang X., Cancel L.M., Tarbell J.M., Khademhosseini A. (2013). Study of long-term viability of endothelial cells for lab-on-a-chip devices. Sens. Actuators B Chem..

[47] Okuyama T., Yamazoe H., Mochizuki N., Khademhosseini A., Suzuki H., Fukuda J. (2010). Preparation of arrays of cell spheroids and spheroid-monolayer cocultures within a microfluidic device. J. Biosci. Bioeng..

[48] Chang J., Shao H., Reiner T., Issadore D., Weissleder R., Lee H. (2012). Microfluidic Cell Sorter (μFCS) for On-chip Capture and Analysis of Single Cells. Adv. Healthc. Mater..

